# A novel point-of-care testing strategy for sexually transmitted infections among pregnant women in high-burden settings: results of a feasibility study in Papua New Guinea

**DOI:** 10.1186/s12879-016-1573-4

**Published:** 2016-06-06

**Authors:** Steven G. Badman, Lisa M. Vallely, Pamela Toliman, Grace Kariwiga, Bomesina Lote, William Pomat, Caroline Holmer, Rebecca Guy, Stanley Luchters, Chris Morgan, Suzanne M. Garland, Sepehr Tabrizi, David Whiley, Stephen J. Rogerson, Glen Mola, Handan Wand, Basil Donovan, Louise Causer, John Kaldor, Andrew Vallely

**Affiliations:** The Kirby Institute, University of New South Wales, Sydney, Australia; Papua New Guinea Institute of Medical Research, Goroka, Papua New Guinea; Department of Obstetrics & Gynaecology, Alotau General Hospital, Milne Bay Province Alotau, Papua New Guinea; Alotau Urban Clinic, Alotau, Milne Bay Province Papua New Guinea; University of Technology, Sydney, Australia; The Burnet Institute, Melbourne, Australia; Melbourne School of Population and Global Health, University of Melbourne, Melbourne, VIC Australia; Department of Microbiology and Infectious Diseases, The Royal Women’s Hospital, Parkville, VIC Australia; Department of Obstetrics and Gynaecology, University of Melbourne, Murdoch Children’s Research Institute, Parkville, VIC Australia; University of Queensland, Herston, QLD Australia; Department of Medicine, University of Melbourne, Melbourne, Australia; Department of Obstetrics & Gynaecology, School of Medicine and Health Sciences, University of Papua New Guinea, National Capital District, Papua New Guinea; Sydney Sexual Health Centre, Sydney, Australia

**Keywords:** Antenatal, Pregnancy outcome, Molecular testing, Syndromic management, Low-middle-income country, Genexpert, Point-of-care, STIs

## Abstract

**Background:**

Sexually transmitted and genital infections in pregnancy are associated with an increased risk of adverse maternal and neonatal health outcomes. High prevalences of sexually transmitted infections have been identified among antenatal attenders in Papua New Guinea. Papua New Guinea has amongst the highest neonatal mortality rates worldwide, with preterm birth and low birth weight major contributors to neonatal mortality. The overall aim of our study was to determine if a novel point-of-care testing and treatment strategy for the sexually transmitted and genital infections *Chlamydia trachomatis* (CT), *Neisseria gonorrhoeae* (NG), *Trichomonas vaginalis* (TV) and Bacterial vaginosis (BV) in pregnancy is feasible in the high-burden, low-income setting of Papua New Guinea.

**Methods:**

Women attending their first antenatal clinic visit were invited to participate. CT/NG and TV were tested using the GeneXpert platform (Cepheid, USA), and BV tested using BVBlue (Gryphus Diagnostics, USA). Participants received same-day test results and antibiotic treatment as indicated. Routine antenatal care including HIV and syphilis screening were provided.

**Results:**

Point-of-care testing was provided to 125/222 (56 %) of women attending routine antenatal care during the three-month study period. Among the 125 women enrolled, the prevalence of CT was 20.0 %; NG, 11.2 %; TV, 37.6 %; and BV, 17.6 %. Over half (67/125, 53.6 %) of women had one or more of these infections. Most women were asymptomatic (71.6 %; 47/67). Women aged 24 years and under were more likely to have one or more STI compared with older women (odds ratio 2.38; 95 % CI: 1.09, 5.21). Most women with an STI received treatment on the same day (83.6 %; 56/67). HIV prevalence was 1.6 % and active syphilis 4.0 %.

**Conclusion:**

Point-of-care STI testing and treatment using a combination of novel, newly-available assays was feasible during routine antenatal care in this setting. This strategy has not previously been evaluated in any setting and offers the potential to transform STI management in pregnancy and to prevent their associated adverse health outcomes.

## Background

Previous studies among antenatal women in low-resource settings have found high prevalences of sexually transmitted and genital infections, particularly *Chlamydia trachomatis* (CT), *Neisseria gonorrhoeae* (NG), *Trichomonas vaginalis* (TV) and Bacterial vaginosis (BV), that are readily curable with cheap antibiotics [1]. Untreated, these bacteria are associated with an increased risk of preterm birth, low birth weight, premature rupture of membranes and other adverse maternal and neonatal health outcomes [1].

Papua New Guinea (PNG) has among the highest neonatal mortality rates worldwide, with an estimated 23 neonatal deaths per 1,000 livebirths [2]. Preterm birth and low birth weight are major contributors to neonatal mortality; in PNG an estimated 16 % of all infants are of low birth weight [3]. In addition, an estimated 15 infants per 1,000 births are stillborn [4]. Preliminary results from the country’s first bio-behavioural survey of STIs in pregnancy (*n* = 765) identified high prevalences of CT (23.1 %), NG (13.8 %), and TV (24.1 %) [5]. Overall, 44 % of pregnant women had at least one of these infections [5]. Around 80 % of women with laboratory-confirmed CT, NG or TV were asymptomatic and therefore would not have been detected and treated using national STI syndromic management guidelines that are based on clinical criteria alone, consistent with WHO recommendations for routine care in low-income settings [6, 7].

The lack of affordable, timely, easy-to-use and accurate STI tests that can be used at point-of-care has meant that for the last two decades routine practice in many low and middle-income countries (LMIC) has been based on syndromic management. Recent advances in STI diagnostics however may herald a new era in STI diagnosis and management in such settings, although this as yet is untested in a LMIC setting such as PNG.

The GeneXpert platform (Cepheid, Sunnyvale CA, USA) provides fully automated, easy-to-use point-of-care molecular tests for CT, NG and TV that are as accurate as laboratory-based nucleic acid amplification tests (NAATs) [8]. This robust and portable platform has already revolutionized the diagnosis and management of tuberculosis in a number of LMIC [9] and is now being used in PNG. Disposable single-use cartridges hold the reagents for the simultaneous detection of CT and NG, with a separate cartridge used for TV detection. Test results are available in 90 (CT/NG) and 60 (TV) minutes, allowing for immediate management.

The platform requires specific training in test procedures, some infrastructure including reliable electrical power or a backup generator, as well as modifications to service provision and patient flow to allow for immediate treatment and contact tracing.

The diagnosis of BV has until recently relied on skilled microscopic examination and scoring of Gram stained vaginal smears using Nugent or Amsel scores. The BVBlue test (OSOM BVBlue Test, Gryphus Diagnostics, USA) is a recently developed chromogenic test for the diagnosis of BV that detects increased vaginal fluid sialidase activity in 10 min. It is the first robust point-of-care test available for BV and has a sensitivity of 90 % and specificity of 95 % compared to Nugent or Amsel scoring [10].

In this paper we present findings from the first feasibility study of antenatal point-of-care testing and treatment of CT, NG, TV and BV using a combination of GeneXpert CT/NG, TV and the BVBlue Test, provided in a routine antenatal clinic setting. This research was conducted in preparation for a large-scale field trial to evaluate this same strategy to improve pregnancy outcomes in PNG and other high-burden, LMIC settings.

## Methods

### Study design and procedures

A descriptive feasibility study was undertaken at a government-run urban antenatal clinic in Alotau, Milne Bay Province, PNG, between August and December 2014. All nursing staff at the clinic (Nursing Officers, Midwives and Community Health Workers) were trained in all aspects of the study, including the point-of-care testing.

Women aged 18 years or over and attending their first antenatal visit were invited to participate. Following written informed consent, women were enrolled consecutively and assigned a unique alphanumeric study identity (ID) number. Nursing staff conducted a short face-to-face interview with each participant, during which socio-demographic and sexual behaviour data were collected.

Women received routine antenatal and provider-initiated HIV and syphilis screening, and haemoglobin estimation. HIV testing was conducted using HIV rapid tests at point-of-care (Alere Determine HIV-1/2; followed by confirmatory Chembio Stat-Pak HIV-1/2), as per national guidelines. Syphilis testing used a rapid point-of-care screening test (SD Bioline anti-TP 3.0, Alere, Germany) followed by a confirmatory laboratory-based rapid plasma reagin (RPR) test. Women with a positive anti-TP plus a reactive RPR (any titre) test were considered to have active syphilis.

Following instructions from the nursing staff, participants provided two self-collected, mid-cavity, vaginal swabs for on-site testing: CT/NG and TV using the GeneXpert platform; and BV using the BVBlue rapid test assay. Testing was conducted by nursing staff supported by the in-country investigator team. Women diagnosed with any of these four infections were offered same-day antibiotic treatment in accordance with PNG STI management guidelines; risk-reduction counselling, and contact tracing was provided as necessary.

### Data management and statistical methods

Study-specific case record forms (CRFs) were used to record socio-demographic, sexual behaviour, obstetric and clinical information. STI test results were automatically recorded in the GeneXpert laptop database by participant study ID number and were similarly recorded in a paper-based laboratory test log. CRF data were entered by study ID into a study-specific database without personal identifiers. Each day, all CRFs and test result log entries were reviewed for completion and consistency by the lead investigators. Clinical and STI test result data were entered into a study-specific Excel database and all electronic database entries validated against participant study folders for accuracy.

Data were summarised as frequencies and percentages. Fisher’s exact test was used to compare statistical differences in outcomes of interest between younger and older women (e.g. STI prevalences). Data were analysed using Stata IC v14.0 (StataCorp LP, Texas, USA). Due to the modest sample size, it was not possible to conduct multivariate analysis to examine independent risk factors.

## Results

Clinic staff were able to conduct point-of-care testing with minimal supervision after one day of intensive training. Interest in the study was high with nearly women who attended antenatal clinic requesting to participate. Due to a clinic schedule that allocates one weekday to new antenatal attendances (resulting in 20–25 new visits on a single day); the availability of limited testing facilities (one single, four-module GeneXpert machine) and the requirement to provide same-day test results, we were unfortunately unable to enrol all those who asked to join. As such, 125 of the 222 (56 %) who attended the clinic were enrolled. Given these limitations women were enrolled as they presented to clinic, i.e. on a first come, first served basis and were not selected in any way by clinic staff or the research team. The integration of study procedures into routine antenatal clinic activities resulted in an average of two hours additional waiting time per woman.

Among the 125 women recruited, the median age was 24 years; around 75 % (81/107) of women attended in the first or second trimester of pregnancy; and almost half were primigravid (59/125; 47 %; Table 1).

**Table 1:** Socio-demographic characteristics, sexual behaviour, obstetric history, genital symptoms and point-of-care test results

		Total	≤24 years	≥25 years	*p* value
		*N* (%)	*N* = 66 (%)	*N* = 59 (%)	
Socio-demographic characteristics					
Age, median (IQR:18-41years)		24 years	21 years	30 years	
Province of birth	Milne Bay Province	100 (80.0)	54 (81.8)	46 (78.0)	
	Other province	25 (20.0)	12 (18.2)	13 (22.0)	
Marital Status	Married	108 (86)	51 (77.3)	57 (96.6)	
	Single	17 (13.6)	15 (22.7)	2 (3.4)	*p* = 0.002
Level of education (*N* = 124)	Never attended school	4 (3.2)	0 (0)	4 (6.7)	*p* = 0.032
	Only primary school	39 (31.2)	18 (27.3)	21 (35.6)	
	Secondary school	81 (65.3)	47 (71.2)	34 (57.6)	
	Tertiary education	24 (19.2)	9 (13.6)	15 (25.4)	
Obstetric history					
Parity (*N* = 124)	Primigravid	59 (47.6)	48 (72.8)	11 (18.6)	
	Para 1–3	46 (37.0)	17 (25.8)	29 (49.2)	*p* < 0.0001
	Para 4 or more	19 (15.3)	0 (0.0)	19 (32.2)	*p* = 0.008
Previous miscarriage/stillbirth/perinatal/neonatal death (*N* = 125)		6 (4.8)	3 (4.5)	3 (5.1)	
Estimated gestational age at enrolment based on LMP (*N* = 107)	12 weeks or less	13 (11.2)	5 (7.6)	8 (13.6)	
	13–26 weeks	68 (63.5)	38 (57.6)	30 (50.1)	
	27–36 weeks	24 (22.4)	9 (13.7)	15 (25.4)	
	More than 36 weeks	2 (1.9)	1 (1.5)	1 (1.7)	
Sexual behaviour					
Age at sexual debut, median (IQR: 13–31years)		19 years	18 years	20 years	
Lifetime number of sexual partners (*N* = 119)	1 partner	37 (31.1)	20 (30.3)	17 (28.8)	
	2–3 partners	51 (42.9)	29 (43.9)	22 (37.2)	
	4–6 partners	21 (17.6)	11 (16.6)	10 (16.9)	
	7 or more partners	10 (8.4)	4 (6.1)	6 (10.2)	
Sexual partners in previous month (*N* = 124)	0	31 (25.0)	18 (27.3)	13 (22.0)	
	1	80 (64.5)	41 (62.1)	39 (66.1)	
	2 or more partners	13 (10.5)	6 (9.1)	7 (11.9)	
Number of times had vaginal sex in the last month (*N* = 122)	0	36 (29.5)	22 (33.3)	14 (23.7)	
	1–2 times	28 (23.0)	11 (16.7)	17 (28.8)	
	3 times or more	32 (26.2)	17 (25.6)	15 (25.4)	
	More than once/unknown	26 (21.3)	15 (22.7)	11 (18.6)	
Condom use last sex (N = 124)	Yes	29 (23.4)	12 (18.2)	17 (28.8)	
	No	95 (76.6)	54 (81.8)	41 (69.5)	
Ever had sex for gifts/favours/money		31 (24.8)	16 (24.2)	15 (25.4)	
Reported genital symptoms at enrolment					
Previous STI/genital infection		28 (22.4)	15 (22.8)	13 (22.0)	
Current genital symptoms	Any symptom	34 (27.2)	20 (30.3)	14 (23.7)	
	Vaginal discharge^a^	24 (19.2)	16 (24.2)	8 (13.6)	
	Genital sores	1 (0.8)	1 (1.5)	0 (0.0)	
	Abdominal pain^a^	8 (6.4)	4 (6.1)	4 (6.8)	
	Itching^a^	19 (15.2)	10 (15.1)	9 (15.3)	
	Other symptoms^b^	6 (4.8)	4 (6.1)	2 (3.4)	
STI test results					
	Any STI	67 (53.6)	42 (63.6)	25 (42.4)	*p* = 0.017
	1 STI	39 (31.2)	22 (33.3)	17 (28.8)	
	2 or more STIs	28 (22.4)	20 (30.3)	8 (13.6)	*p* = 0.025
	HIV	2 (1.6)	2 (3.0)	0 (0.0)	
	Active syphilis^c^	5 (4.0)	3 (4.5)	2 (3,4)	
	*C. trachomatis* (CT)	25 (20)	18 (27.3)	7 (11.9)	*p* = 0.032
	*N. gonorrhoeae* (NG)	14 (11)	12 (18.2)	2 (3.4)	*p* = 0.009
	*T. vaginalis* (TV)	47 (37.6)	31 (47.0)	16 (27.1)	p = 0.022
	Bacterial vaginosis (BV)	22 (17.6)	11 (16.7)	11 (18.7)	
	Genital symptoms and 1 or more STI detected	19 (15.2)	16 (24.3)	3 (5.1)	*p* = 0.002
	Genital symptoms but no STI detected	15 (12.0)	4 (6.1)	11 (18.6)	*p* = 0.031

^a^14 women had multiple symptoms/^b^ discharge, pus, “wetness”/^c^anti-TP+ and RPR+, all titres

Province of birth was considered significant so as to identify what proportion of women had been born in the study province (Milne Bay) and what proportion were not, in order to look at differences in STI prevalence among these groups. ‘Other province’ includes all other provinces in PNG (there are 22 in total). Due to small numbers for each individual province we aggregated these and present this as ‘Other province’ rather than individual provinces.

Six women (6/125; 5 %) had a previous history of miscarriage, stillbirth or neonatal death. Based on point-of-care test results, more than half of women (67/125; 53.6 %) had any of CT, NG, TV or BV, among whom 71.6 % (48/67) were asymptomatic. Overall, the prevalence of CT was 20.0 %; NG, 11.2 %; TV, 37.6 %; and BV, 17.6 %. The prevalence of HIV was 1.6 %, and 4.0 % of the women had active syphilis. Women aged 24 years or under were significantly more likely to have any of CT, NG, TV or BV, compared with older women (63.6 % vs. 42.4 %; odds ratio [OR] 2.38, 95 % confidence interval [CI]: 1.09, 5.21). Younger women with genital symptoms were six times more likely to have an infection compared with older women who had genital symptoms (25.8 % vs. 5.1 %; OR 6.48, 95 % CI: 1.70, 36.0). There were no significant differences between younger and older women in sexual behavioural characteristics, past history of an STI, or current genital symptoms. Among the 67 women who had one or more of CT, NG, TV or BV, there were no significant differences in sexual behavioural characteristics between younger and older women.

Among the 34 women who reported genital symptoms at enrolment, around half (19/34; 55.9 %) had any of CT, NG, TV or BV. Among the six women who reported a previous adverse pregnancy outcome, four had a history of stillbirth, of whom two had TV, and one had CT, TV and BV in the current study. All women received their test results the same day. Of those with any STI or genital infection, 83.6 % (56/67) received same-day treatment.

The remaining 12.4 % (11/67) had to leave prior to receiving treatment due to family commitments or the need to travel significant distances back to their homes by foot or by bus. All women had received treatment within one week. Partners in attendance at the clinic were treated the same day. All women and their partners diagnosed with an infection received treatment in accordance with PNG national guidelines [11]. Given the resource limitations of this study, test of cure follow-up was not possible. It will however be a part of future large scale antenatal trials.

## Discussion

Antenatal point-of-care STI testing and treatment using a combination of novel, newly-available diagnostic tests proved feasible when integrated as part of routine antenatal care in this setting. This is the first study in any setting to evaluate the feasibility of this approach. The high interest shown in study participation and 100 % completion of study procedures among those enrolled suggest the intervention has a high level of acceptability, but needs to be formally established. An important limitation was our inability to meet antenatal testing demand: we were only able to recruit around 56 % of those who attended yet nearly all women expressed an interest in joining the study. Future intervention studies and demonstration projects should consider setting up more than one four-module GeneXpert machine per clinic in situations of high workload.

High prevalences of STIs and genital infections were observed among pregnant women in this setting, particularly those aged 24 years or under. These findings are consistent with our earlier research among antenatal women in PNG [12, 13]. A minority of women (34/125; 27.2 %) in the current study reported genital symptoms at enrolment, of whom around half had CT, NG, TV or BV; whilst more than 70 % of women with one or more of these curable infections were asymptomatic. These findings suggest that STI syndromic management, based on clinical presentation alone, is a poor strategy for the detection and treatment of STIs among pregnant women in this setting and underline the need for robust, easy-to-use, timely, and accurate point-of-care tests.

In this pilot study, the cost-effectiveness of this new and broad based POC testing approach was not examined in detail. A cluster randomised trial of antenatal point-of-care testing and treatment to improve pregnancy outcomes is due to start in PNG in 2016 [13]. This trial will establish the efficacy, acceptability, cost-effectiveness and health system requirements of the proposed test and treat strategy, and will inform future international policy and practice on the diagnosis and management of STIs in pregnancy.

## Conclusions

With increased Genexpert device availability this integrated point of care testing strategy has the potential to change the way STI’s in pregnancy are managed and reduce the number of associated adverse health outcomes for antenatal women in Papua New Guinea.

## Abbreviations

BV, Bacterial vaginosis; CT, Chlamydia trachomatis; CRF, Case record form; HIV, Human Immunodeficiency Virus; LMIC, Low middle income country; NAAT, Nucleic acid amplification test; NHMRC, National Health and Medical Research Council; NG, Neisseria gonorrhoeae; PNG, Papua New Guinea; POC, Point of care; RPR, Rapid plasma reagin; STI, Sexually transmitted infection; TV, Trichomonas vaginalis; WHO, World Health Organisation
